# Lack of thermal acclimation in multiple indices of climate vulnerability in bumblebees

**DOI:** 10.1098/rspb.2024.2216

**Published:** 2025-01-15

**Authors:** C. L. Poore, E. J. Ibarra-Garibay, A. L. Toth, E. A. Riddell

**Affiliations:** ^1^ Department of Ecology, Evolution, and Organismal Biology, Iowa State University, 2200 Osborn Drive, Ames, IA 50010, USA; ^2^ Department of Biology, University of North Carolina, Chapel Hill, NC 27514, USA

**Keywords:** climate vulnerability, metabolic rate, water loss rate, survival, thermal tolerance, *Bombus*

## Abstract

Indices of climate vulnerability are used to predict species’ vulnerability to climate change based on intrinsic physiological traits, such as thermal tolerance, thermal sensitivity and thermal acclimation, but rarely is the consistency among indices evaluated simultaneously. We compared the thermal physiology of queen bumblebees between a species experiencing local declines (*Bombus auricomus*) and a species exhibiting continent-wide increases (*B. impatiens*). We conducted a multi-week acclimation experiment under simulated climate warming to measure critical thermal maximum (CT_max_), critical thermal minimum (CT_min_), the thermal sensitivity of metabolic rate and water loss rate and acclimation in each of these traits. We also measured survival throughout the experiment and after the thermal tolerance trials. Neither species acclimated to the temperature treatments by adjusting any physiological trait. We found conflicting patterns among indices of vulnerability within and between species. We also found that individuals with the highest CT_max_ exhibited the lowest survival following the thermal tolerance trial. Our study highlights inconsistent patterns across multiple indices of climate vulnerability within and between species, indicating that physiological studies measuring only one index of climate vulnerability may be limited in their ability to inform species’ responses to environmental change.

## 1. Introduction

Climate change has had severe negative effects on the persistence of populations and species over the last few decades [1–3], but the underlying mechanisms driving species responses to warming remain complex and challenging to identify [4,5]. Several organismal traits have been proposed as indices of climate vulnerability [6–8], and these typically relate to the capacity of the organism to physiologically tolerate environmental change, particularly with regard to temperature. These traits describe the temperature at which organisms lose motor control, the rate at which they consume energy with respect to temperature, and their capacity to adjust fitness-related traits after exposure to temperatures [9]. Many of these indices are based on the concept of the thermal performance curve [10], which is often used in ectotherms to describe the nonlinear relationship between body temperature and an aspect of performance or fitness [11]. Indices developed from thermal performance curves have shaped our modern understanding of climate vulnerability in ectotherms across the globe [11,12], but whether the suite of indices derived from thermal performance curves reflects consistent patterns of climate vulnerability remains to be understood.

Thermal performance curves are bounded by thermal tolerances, which are referred to as the critical thermal minimum (CT_min_) for the lower limit and the critical thermal maximum (CT_max_) for the upper limit [11]. These indices are often measured in experiments as the temperature at which coordination or performance is severely reduced [13,14]. In theory, CT_max_ is not supposed to be lethal; however, these experiments are often associated with high mortality in insects [15]. By combining critical thermal tolerances with estimates of field body temperatures, studies have used thermal tolerances to predict the ecological impacts of climate change on a wide range of terrestrial and marine ectotherms [6,12,16–19]. Recent empirical studies have demonstrated that variation in CT_max_ at the species or individual level may be associated with survival [20,21]; however, thermal limits should be interpreted cautiously due to the effects of methodology and inconsistent definitions [22]. Thermal performance curves also describe the thermal sensitivity of performance, which characterizes the rate at which performance in ectotherms changes with temperature [23]. Therefore, ectotherms with greater thermal tolerances and lower thermal sensitivity are expected to be less vulnerable to warming. Importantly, these traits are not static and can change with exposure to environmental change, which provides an additional axis of climate vulnerability.

Organisms can shift the relationship between performance and temperature through a process known as thermal acclimation, whereby organisms adjust thermal tolerance or thermal sensitivity in ways that benefit performance or fitness in a new thermal environment [16,24]. The acclimation capacity of organisms has been proposed as an important index of vulnerability to climate change [7,8] because species with greater acclimation capacities can shift thermal performance closer towards the new trait optimum in warmer environments [25]. Models that incorporate thermal acclimation of physiological traits (e.g. water loss rates and metabolic rates) support the role of thermal acclimation in reducing climate vulnerability [26–28]. Metabolic rates are particularly relevant for organismal performance because they influence energy budgets, competitive ability, growth rates and lifetime reproductive success [29,30]. Thus, organisms with a high thermal sensitivity of metabolism may rapidly deplete energy reserves at warmer temperatures [27], leading to lower fitness. Similarly, water loss rates influence the risk of desiccation, duration of activity (such as foraging) and the ability to avoid overheating via evaporative cooling [31,32]. Although many studies examine indices of vulnerability independently, to our knowledge, no studies have simultaneously compared acclimation in these traits to other indices of vulnerability. Comparing indices of climate vulnerability can determine whether aspects of thermal performance curves provide consistent patterns of vulnerability, which may arise because selection favours wider tolerance breadths (i.e. greater thermal tolerances), lower thermal sensitivity and greater acclimation capacity in ectotherms that perform better in more variable environments [33–35]. However, these traits may also evolve independently [23], making each index context-specific. Nevertheless, species with greater thermal tolerance, low thermal sensitivity and high acclimation capacity are generally considered to be less vulnerable to environmental change.

In this study, we compared multiple indices of climate vulnerability between two species of North American bumblebees, the common eastern bumblebee (*Bombus impatiens*) and the black and gold bumblebee (*B. auricomus*). Bumblebees are ecologically and economically important pollinators of wild and agricultural plants [36], yet some species are currently experiencing dramatic declines in North America [37–39]. Some declines have been associated with climate change, but the responses between species are highly variable [40,41]. We chose to study a widely distributed and abundant species (*B. impatiens*) that is native to Iowa and has become increasingly common, while also recently expanding its geographical range [39,42]. In the present study, we examined locally collected *B. impatiens*, but we note the species is also commercially reared for pollination, which has contributed to their range expansion in the Pacific Northwest [43]. We also studied a less common Iowa-native species, *B. auricomus* [44,45], which persists in only 50% of its historic range [46,47]. We fully recognize that a two-species study does not test whether variation in physiology drives climate vulnerability in bumblebees in general [48]. Rather, we used these demographic patterns to generate hypotheses on interspecific differences in climate vulnerability and explore consistency among indices of climate vulnerability.

We conducted a multi-week acclimation experiment on individual queen bumblebees to measure thermal tolerance, thermal sensitivity of metabolic rate and water loss rate and acclimation capacity of these traits. Queen bumblebees were chosen as focal individuals because they have relatively longer lifespans than workers or males (and thus more potential for acclimation), spend most of their life outside the social colony environment and determine the effective population size of bumblebees [49]. Because queens establish nests and are active across a broad range of environmental conditions [50], individual queens may shape ecological responses of species to changing environments. However, queens also spend most of their life underground [50], which may buffer them from temperature variation and the need to acclimate. Previous experiments on *B. impatiens* suggest a limited capacity for acclimation of thermal tolerances [51,52]. However, these experiments were conducted on workers over short periods of time (12–72 h) under stable temperatures. Here, we studied the responses of several physiological traits to ecologically realistic (and physiologically challenging) fluctuations in temperature under current and future warming scenarios for multiple weeks. We also measured survival throughout the experiment and following the thermal tolerance experiments to compare survival outcomes between species and determine whether any indices of climate vulnerability were related to survival. Based on observed demographic trends, we hypothesized that *B. impatiens* would exhibit greater thermal tolerances, lower thermal sensitivity and higher acclimation capacities compared with *B. auricomus*—all of which would indicate potential resilience in the face of environmental warming. By combining experiments on multiple physiological traits through time, our study has the potential to provide a more complete understanding of species’ vulnerability to a warming planet.

## 2. Methods

### (a) Collection and care

We collected wild bumblebee queens of *B. impatiens* (*n* = 43) and *B. auricomus* (*n* = 38) by net between 4 May and 24 May 2021 from sites in Ames and Coon Rapids, Iowa. Both species were represented at each site (*B. impatiens*: Ames = 31, Coon Rapids = 12; *B. auricomus*: Ames = 23, Coon Rapids = 15). In Coon Rapids, we captured all bees from the Whiterock Conservancy Nature Preserve with permission from the preserve, and in Ames, we collected on private property, local parks in Ames and Iowa State University campus with permission from landowners and the university. These sites are approximately 111 km apart, with Coon Rapids being more rural than Ames. The activity of queens is highest during May for both species [50], suggesting these two species have similar phenology and are thus at similar life stages when collected. We transferred queens to 50 ml ventilated Eppendorf tubes, assigned each individual a unique identifier consisting of species and specimen number and placed each tube in a cooler on ice, during which time the bees entered a chill coma. This method is commonly used as a non-lethal method of immobilizing insects for research and did not have any clear connections with mortality in our experiment (see §3e). Within 4 h from when the individuals were collected, we transported the queens to the laboratory at Iowa State University.

Upon returning to the lab, we weighed each individual to the nearest 0.001 g. We immediately transferred queens to individual, clear plastic enclosures (described in [53]; Country Plastics, Ames, IA, USA) and placed them in an environmental control chamber (Percival Scientific, Perry, IA, USA) with a cycling temperature and humidity regime designed to simulate current climatic conditions in the field (see §2b). We monitored individuals at least once daily for two weeks (14 ± 1 days) and provided 50% (w/v) sucrose solution ad libitum prior to initial measurements. We also provided balls of pollen (stored frozen; Stakich, Troy, MI, USA) mixed with sucrose solution every two days [54]. We removed any brood cells throughout the study to minimize effects of brood rearing on queen physiology, which only occurred in five individuals (three *B. auricomus* and two *B. impatiens*). We also removed pollen two days prior to respirometry measurements to minimize pollen residue in the respirometry chambers.

### (b) Temperature treatments

We used walk-in environmental chambers to simulate fluctuating temperature regimes. Every individual experienced a current climate scenario during the lab acclimation period (i.e. prior to the initial physiological measurements). After the initial physiological measurements, bees were randomly assigned to return to the current scenario (as a control) or experience a warming scenario (see electronic supplementary material, figure S1 for an overview of the experiment). Each room operated under a 24 h cycle of temperature, humidity and light (electronic supplementary material). We created the temperature cycle (electronic supplementary material, figure S2) for these treatments based upon estimates of operative temperatures (*T*_e_) that bumblebees experience on an average day in the spring in Ames, IA while active in the sun during the day and nesting belowground at night (electronic supplementary material, section S1). We also verified that our estimates of operative temperature were accurate (electronic supplementary material, figure S3, section S2). For the climate warming treatment, we increased the air temperatures by 4°C to simulate warming based on the expected increase in air temperature under the Representative Concentration Pathway 8.5 (RCP 8.5) throughout much of North America (IPCC 2021). The warming treatment was expected to elicit acclimation because bumblebees become physiologically stressed at temperatures between 32°C and 35°C, leading to 100% mortality when reared at 35°C after one week [55]. Similarly, flight performance begins to decline dramatically after 30°C [56]. In our warming treatment, maximum temperatures reached 32.5°C, compared with 28.5°C in the current climate scenario. Thus, the climate warming treatment should be more physiologically stressful compared with the current climate treatment. In both treatments, relative humidity was regulated to maintain a constant vapour pressure deficit (VPD) of 0.95 kPa, in which the VPD refers to the evaporative demand of the air. By controlling the evaporative demand of the air, the treatments were designed to specifically evaluate physiological acclimation in response to temperature. We note that instances of endothermy may influence the true water vapour density gradient experienced by an individual [57,58], but regardless, the bees still experienced similar evaporative demands of the air with respect to treatment. We also verified these conditions were relevant using ten years of hourly data from a local weather station at one of our collection sites. Although VPDs are generally higher during the day at the field site, our treatments are still ecologically relevant because VPDs are at least 1.0 kPa at all times of day in the late spring and early summer (electronic supplementary material, figure S4). The lights in the chamber also simulated semi-natural conditions by turning on at 06.00 at 10% intensity, reaching 100% intensity by 1000 and turning off by 2000 in both treatments.

### (c) Metabolic and water loss measurements

After acclimating to laboratory conditions in the current climate treatment room for 14 ± 1 days, we removed up to seven queens from their individual enclosures between 1700 and 1745 and transferred them to a glass respirometry chamber (length: 59.5 mm; inner diameter: 32.0 mm; thickness: 2.0 mm). We also measured non-shivering thermogenesis 24 h prior to respirometry trials, which is beyond the scope of the study and unlikely to affect our results (electronic supplementary material, section S3). They were then moved from their environmental chamber to a reach-in incubator to measure the thermal sensitivity of physiological rates at rest overnight from 1700 to 0900 (see electronic supplementary material, section S4 for details on the respirometry system). We conducted these measurements at night to ensure that bees were at rest and inactive, which follows standard protocol for measuring standard metabolic rates in ectotherms [59]. We acknowledge that metabolic rates may change throughout the day due to activity and circadian rhythm effects [60], which may affect our interpretations of acclimation. We measured metabolic rate as the volume of carbon dioxide production (*V*CO_2_) and evaporative water loss rates (EWL) at two temperatures (30°C and 18°C) on separate nights, which we selected based on conditions they regularly experience in nests (30°C) [61] and during activity outside the nest (18°C) [56,62]. We measured each individual at the warmer temperature first followed by the cooler treatment, providing approximately 30 h between measurements for rest in their individual boxes. To account for the different lengths of gas exchange cycles between temperature treatments, we measured each chamber for 100 min at 18°C and 40 min at 30°C [63]. Using these measurement periods, we recorded an average of 4.87 ± 3.61 (mean ± standard deviation) discontinuous gas exchange (DGE) cycles per individual at 18°C and 17.03 ± 13.03 cycles at 30°C. In total, we collected 3128 respiration measurements across all treatments and species. At 30°C, we used the DG-4 to regulate the humidity at 2.200 kPa (±0.010 kPa), which created a VPD of approximately 2.0 kPa. At 18°C, we regulated the DG-4 at 1.000 kPa (±0.010 kPa), which created a VPD of approximately 1.0 kPa (see below for analysis on vapour pressure deficits). In the statistical analysis, we disentangle the confounding effects of temperature and VPD by using a VPD-corrected evaporative water loss rate, which accounts for differences in VPD [64].

We used Expedata (v. 1.9.27, SSI) to collect and process raw respirometry data (electronic supplementary material, section S5). In addition to measuring the average metabolic and water loss rates, we also measured the duration of each gas exchange cycle (which is inversely related to the frequency) and the total volume of each breath to compare patterns between species and treatments. At rest, many insects (including bumblebees) exhibit DGE, which is defined as periodic exchange (or bursts) of CO_2_ and O_2_. The frequency of DGE cycles generally increases with ambient temperature, and at high enough temperatures, DGE gives way to cyclic gas exchange (in which CO_2_ levels fail to return to zero in between gas exchange cycles) and then continuous gas exchange (in which spiracles remain fully open [65,66]). In our study, the presence of DGE indicated that individuals were at rest (and thus ectothermic) during measurements [63]. We only measured metabolic rates when DGE or patterns of cyclical breathing were observed (which occurred at the warmer temperature). Although patterns of cyclical breathing are not strictly DGE, bees were still likely ectothermic because metabolic rates of endothermic bees are nearly 10-fold higher than rates observed in our study [67]. Regardless of the pattern, we measured *V*CO_2_ from peak to peak (avoiding any irregular peaks) following standard protocols (see electronic supplementary material for description). We did not include any measurements of continuous breathing, which were easily identifiable and typically indicative of activity (electronic supplementary material, figure S5).

Following the initial measurements, individuals were randomly assigned to the current climate treatment or climate warming treatment with respect to species. After 21 days of acclimating to the treatments, we measured the queens again at both temperatures (18°C and 30°C) to quantify the acclimation capacities of *B. impatiens* and *B. auricomus*. These measurements took place under the same conditions and in the same order as the pre-acclimation measurements: first an overnight measurement at 30°C, then an approximately 30 h recovery period and finally an overnight measurement at 18°C.

### (d) Critical thermal limits

Two days following the last post-acclimation metabolic measurement, we measured thermal tolerances for each individual using a custom apparatus inspired by Oyen & Dillon [51]. We placed an aluminium plate with wells (43.0 mm diameter wells, with a small notch to fit a temperature probe) on a thermoelectric plate (CP-200-HT-TT, TE Technology, Traverse City, MI, USA) connected to a PELT-5 temperature controller (Sable Systems International, Las Vegas, NV, USA) in a reach-in incubator (I-36VL, Percival Scientific, Perry, IA, USA) programmed to 25°C. We coated the inside of glass cylindrical chambers (length: 48.2 mm; outer diameter: 42.0 mm; thickness: 3.2 mm) with INSECT-a-SLIP (BioQuip, Rancho Dominguez, CA, USA) and placed them into the wells. To measure temperature, we drilled a notch in each well that housed a type T thermocouple in contact with the aluminium and the exterior of the glass cylinder. Temperatures for each well were tracked in real time using a TC-08 thermocouple data logger (Pico Technology, Tyler, TX, USA). We measured up to seven queens in individual glass chambers and placed a rubber stopper on top of the cylinder to prevent escape. Air circulated into the chamber during the experiment through a hole in the rubber stopper. Every 4 min, we adjusted the air temperature in the chamber to closely match the temperature regulated by the PELT-5 system to minimize any boundary layer effects that might cause temperature gradients.

We monitored individuals for behaviours indicative of critical thermal limits (described in [51]). For CT_min_ measurements, the PELT-5 temperature controller was programmed to hold the queens at 25°C for 10 min before beginning a ramp to −5°C at a rate of –0.25°C min^−1^. Beginning at 10°C, we assessed responsiveness with an external stimulus by touching individuals on the thorax every 2 min with a haematocrit tube [68]. When individuals displayed curling behaviour indicative of CT_min_, we also verified that individuals failed to respond to stimuli. We then recorded the temperature and immediately removed queens from the aluminium plate and returned them to their enclosures. They were allowed to recover from CT_min_ on the bench top (approx. 22°C), and when all bees reached CT_min_, they were returned to their respective treatment rooms. We allowed individuals to recover from CT_min_ trials for two days before CT_max_ trials. For CT_max_, bees were held at 25°C for 10 min before ramping to 55°C at a rate of +0.25°C min^-1^. We recorded CT_max_ as the temperature at which individuals began involuntarily spasming and postural control began to fail [14,51]. Upon reaching CT_max_, we immediately removed bees from the aluminium plate and returned them to their enclosures to recover on the bench top (approx. 22°C). After all queens reached CT_max_, they were returned to their respective treatment rooms. We minimized potential effects of body size (i.e. differences in thermal inertia) by using a slow ramping rate and matching the air temperature with plate temperature to avoid boundary layer effects. Although *B. auricomus* were larger than *B. impatiens* (average mass = 0.981 g compared with 0.681 g, respectively), the difference in mass would be unlikely to produce substantial differences in body temperatures due to thermal inertia [69].

### (e) Survival

We monitored survival throughout the entire experiment and at four time periods after the thermal tolerance trials. We specifically measured survival for 24 h after the CT_min_ trial and 3 , 24, 48 and 72 h after the CT_max_ thermal tolerance trials. After 72 h remaining live queens were euthanized by chilling overnight in a freezer.

### (f) Statistical analyses: mass, metabolic rate and water loss

We conducted all statistical analyses using R (v. 4.1.1) and conducted separate analyses on each physiological trait (i.e. *V*CO_2_, duration of gas exchange cycle, total volume of gas exchanged, EWL). We do not provide the results for *V*O_2_ because they were not qualitatively different from (and are functionally equivalent to) the *V*CO_2_ results. We exported all results from *R* into Datagraph for visualization.

We analysed each trait separately using linear mixed-effects models (lmer) from the *lme4* package [70] and included individuals as a random effect to account for repeated measures on individuals. To test whether mass varied across the experiment, we assessed differences in body mass prior to initial and final respirometry measurements by including species, acclimation and their interaction as factors in the analysis. For all respirometry data, we averaged across the values for each individual at a given temperature and acclimation time point (pre-acclimation or post-acclimation) using the *plyr* library [71] to avoid statistical artefacts related to having a large sample size. For each analysis, we included temperature (18°C or 30°C), species (*B. impatiens* or *B. auricomus*) and acclimation treatment (pre-acclimation, post-acclimation current or post-acclimation future) and their interactions as fixed categorical effects.

For each physiological trait, we also included mass (the final mass measurement before the last respirometry trial) as a covariate in the analysis to account for the effects of body size [72]. Including mass as a covariate assumes that the effect of mass does not differ between species [73]; thus, for each analysis, we explicitly tested for an interaction between species and mass on each dependent variable in a separate analysis. None of these interactions were significant (*p* ≥ 0.61), indicating that the effect of mass did not vary between species. We also explored the effect of site by including an interaction between site (Ames or Coon Rapids) and species; however, we had no *a priori* predictions for site-level differences and removed site from the analysis when non-significant [74]. We ensured each model met the assumptions of normality and heteroscedasticity for linear regressions by assessing Q–Q plots and the residuals, respectively. We log-transformed the duration of the *V*CO_2_ cycle to meet the assumption of normality. We also assessed collinearity using variance inflation factors (GVIF^1/(2*df)^) [75] and ensured values did not exceed a threshold of 10 (or 3.16 if when using GVIF^1/(2*df)^) [76]. We then performed a type II analysis of covariance (ANCOVA) using the lmerTest() library to identify significant variables and interactions [77].

### (g) Statistical analyses: thermal tolerance

For the thermal tolerance trials, we used linear models (*lm*) to analyse CT_min_ and CT_max_ in separate analyses. We also separately analysed the thermal tolerance breadth (TTB) by subtracting CT_min_ from CT_max_ for each individual. In each model, we included species, acclimation treatment (current or future), body mass and the interaction between species and acclimation treatment. We also assessed the effect of site (as above). We conducted a type II ANCOVA using the *car* package to analyse the significance of each variable and the interaction effect between species and acclimation treatment. We also determined whether the results were robust to outliers by removing three statistical outliers from *B. impatiens*.

### (h) Statistical analyses: survival

For the analysis comparing survival between species, we used a Cox proportional hazard model from the *survival* package [78], which included species and treatment as factors and body mass as a covariate. We also ran individual models with each independent variable to determine whether the results were due to a lack of statistical power from overfitting. We determined whether individual variation in indices of vulnerability was correlated with survival using logistic regressions to assess the relationship between survival three days after the thermal tolerance trials. Species was included as a factor, and we assessed the effect of mass using the same procedure as above.

### (i) Statistical analyses: correlations between indices

To assess correlations between indices of climate vulnerability, we used Pearson’s correlation coefficients to assess the association between trait indices at the individual level and used *t*-tests to assess significance. We conducted each test separately on each species. We also adjusted trait values in any analysis that assessed the thermal sensitivity of a trait and the change in the thermal sensitivity of the trait (i.e. acclimation) to avoid statistical artefacts related to regression to the mean [79]. If acclimation was significant, we used post hoc analyses (Tukey HSD) to determine specifically which treatment groups were statistically different from one another.

## 3. Results

### (a) Body size

Body mass for *B. auricomus* and *B. impatiens* was 0.981 ± 0.003 g and 0.681 ± 0.003 g, respectively. Body mass was significantly larger in *B. auricomus* queens compared with *B. impatiens* queens (F_1, 105_ *=* 669.13, *p* < 0.001), and body mass was not significantly different between acclimation treatments (F_2, 97_ *=* 0.223, *p =* 0.801) or by the interaction between species and acclimation treatment (F_2, 97_ = 1.904, *p =* 0.154), indicating that queens of both species maintained mass during the experiment.

### (b) Standard metabolic rate

**Figure 1.**
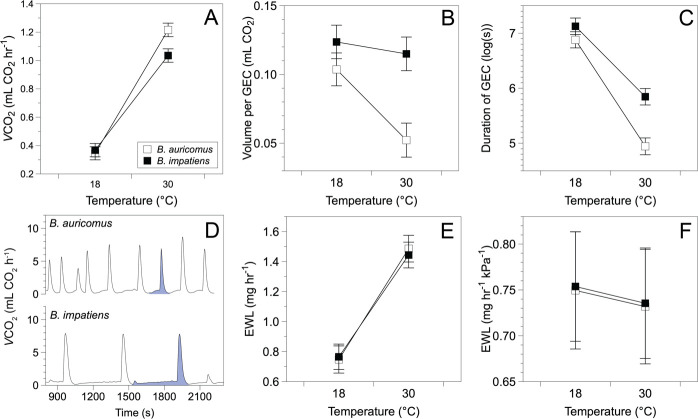
Physiological differences between *B. impatiens* and *B. auricomus.* (A) *B. auricomus* exhibited a higher metabolic rate than *B. impatiens* at 30°C, indicating greater thermal sensitivity in *B. auricomus.* (B,C) *B. auricomus* also exhibited (B) shallower and (C) quicker gas exchange cycles (GEC) relative to *B. impatiens*, particularly at warmer temperatures. (D) Gas exchange cycles for *B. auricomus* and *B. impatiens* at 30°C. A single cycle is highlighted in purple. (E) *B. auricomus* and *B. impatiens* exhibited similar water loss rates and (F) VPD-corrected rates of water loss at both temperatures, indicating similar thermal sensitivity. Values account for differences in body size, and estimated marginal means are plotted with 95% confidence intervals.

Queens of both species displayed respiratory patterns at rest that were characteristic of DGE (electronic supplementary material, figure S6). *V*CO_2_ rates increased with temperature for both species (F_1, 196_ *=* 1568.2, *p* < 0.001) but the effect of temperature differed by species (F_1, 196_ *=* 27.6, *p* < 0.001) with *B. auricomus* exhibiting greater thermal sensitivity than *B. impatiens* (figure 1A). At 18°C, *B. impatiens* and *B. auricomus* exhibited similar rates of *V*CO_2_, but at 30°C, *V*CO_2_ was 9.2% higher in *B. auricomus* than *B. impatiens*. Body mass was also positively associated with *V*CO_2_ (F_1, 68_ *=* 6.9, *p* = 0.011). *V*CO_2_ did not change with species (F_1, 66 _*=* 1.4, *p =* 0.245) or acclimation treatment (*F_2, 212_ =* 1.5, *p =* 0.231). We also did not find significant effects of acclimation and its interaction with temperature (F_2, 196_ = 1.8, *p =* 0.162), species (F_1, 211_ *=* 1.7, *p =* 0.190) or temperature and species (F_2, 195_ *=* 0.1, *p =* 0.892). We also found that *B. auricomus* took shallower and more frequent breaths at warm temperatures relative to *B. impatiens* (figure 1B,C,D; electronic supplementary material, section S6).

### (c) Evaporative water loss rates

Rates of EWL were significantly higher at 30°C compared to rates at 18°C (figure 1E; F_1, 161_ = 792.1, *p* < 0.001). Body mass also exhibited a significant positive relationship with EWL rate (F_1, 68_ = 5.01, *p =* 0.029), and individuals collected at Coon Rapids had significantly higher EWL compared to individuals from Ames (F_1, 65_ = 22.8, *p* < 0.001). The site effect was similar for each species (F_1, 64_ = 1.4, *p* = 0.242). There were no significant effects of species (F_1, 64_ = 0.5, *p =* 0.483) or acclimation treatment (F_2, 184_ = 0.23, *p =* 0.791), nor were there significant interactions between temperature and species (F_1, 162_ = 1.4, *p =* 0.246), temperature and acclimation treatment (F_2, 161_ = 0.7, *p =* 0.500), species and acclimation treatment (F_2, 184_ = 1842.0, *p =* 0.132) or the three-way interaction of temperature, species and acclimation treatment (F_2, 161_ = 0.3, *p =* 0.769). In addition, most of the water loss was likely cuticular, rather than respiratory (electronic supplementary material, figure S7). Because temperature was not a significant predictor of EWL rates after VPD-correction (figure 1F; F_1, 164_ = 0.8, *p =* 0.366), the analysis indicates that EWL was likely driven by humidity and not temperature (electronic supplementary material, section S6).

### (d) Critical thermal limits and breadth

**Figure 2.**
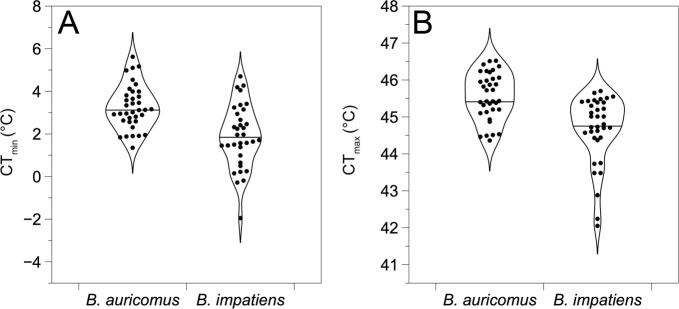
Thermal tolerances of *B. auricomus* and *B. impatiens*. (A) *B. impatiens* had a lower CT_min_ than *B. auricomus*, and (B) *B. auricomus* had a higher CT_max_ than *B. impatiens*. Violin plots are shown with individual measurements (solid points) and the median value (solid black horizontal line) for each trait and species.

Queens became more sluggish and less responsive to stimulus as temperatures cooled, ultimately exhibiting abdominal curling consistent with CT_min_. *B. impatiens* exhibited a lower CT_min_ (mean ± standard error = 1.72 ± 0.25°C) compared to *B. auricomus* (mean ± standard error = 3.43 ± 0.24°C) (F_1,64_ *=* 17.3, *p* < 0.001) (figure 2A). Body mass (F_1, 64_ *=* 2.4, *p =* 0.124), acclimation treatment (F_1, 64_ *=* 1.9, *p =* 0.175) and the interaction between acclimation treatment and species (F_1, 64_ *=* 0.2, *p =* 0.684) did not affect CT_min_. For CT_max_, we noted the onset of muscle spasms and wing fluttering as well as difficulty maintaining postural control. The CT_max_ of *B. impatiens* (mean ± standard error = 44.58 ± 0.16°C) was also lower than *B. auricomus* (mean ± standard error = 45.63 ± 0.16°C) (figure 2B; F_1, 65_ *=* 8.6, *p* = 0.004). Neither body mass (F_1, 65_* <* 0.1, *p =* 0.908), acclimation treatment (F_1,65_ = 0.1, *p =* 0.740) nor the interaction between species and acclimation treatment (F_1, 65_ *=* 0.8, *p =* 0.361) were significant model terms. The effect of species was still significant after removing three outliers from *B. impatiens* (F_1, 62_ *=* 5.6, *p* = 0.021).

Although CT_min_ and CT_max_ differed significantly across species, the overall breadth of thermal tolerance (mean ± standard error = 42.52 ± 0.190°C) did not significantly differ between species (F_1, 64_ *=* 3.38, *p =* 0.07). Moreover, the breadth of thermal tolerance was not significantly predicted by mass (F_1, 64_ *=* 1.7, *p =* 0.193), acclimation treatment (F_1, 64_ *=* 1.6, *p =* 0.207) nor the interaction between species and acclimation treatment (F_1, 64_ < 0.1, *p =* 0.888).

### (e) Survival

During the experiment, 94.7% of *B. auricomus* (36 of 38) and 81.2% of *B. impatiens* (35 of 43) survived from the first metabolic measurement to the thermal tolerance trials, which occurred over approximately 22 to 23 days. Nearly 99% of queens (70 of 71) survived CT_min_ treatments before beginning CT_max_ trials. All 70 queens that began CT_max_ trials survived for at least 3 h following the completion of the trial. By 72 h after the thermal tolerance trial, 48.6% of *B. auricomus* were alive, compared to 74.3% of *B. impatiens*. In the survival model with species, acclimation and body mass, none of the variables were statistically significant (*p* > 0.4). However, in models with only one response variable, we found a trend towards differences in survival between species (*Z* = 1.8; *p* = 0.071), but not body mass (*Z* = −1.3; *p* = 0.13) or acclimation treatment (*Z* = −0.8; *p* = 0.405).

### (f) Correlations with survival and between indices

**Figure 3.**
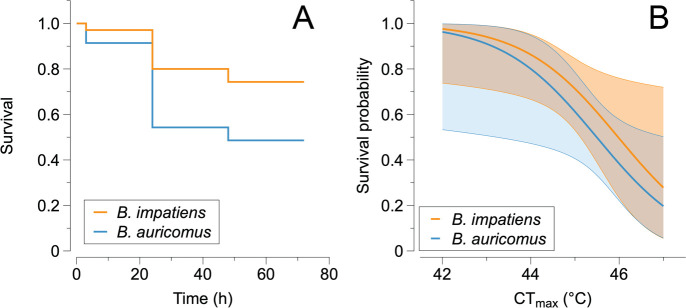
The relationships between survival and thermal tolerance between species. (A) Survival declined more sharply for *B. auricomus* (blue) compared to *B. impatiens* (orange) over a period of three days after the thermal tolerance trials. (B) Individuals with higher CT_max_ exhibited a lower probability of survival in the days following the thermal tolerance trial for both species, suggesting that survival is a product of exposure to stressful temperatures (rather than a species’ intrinsic thermal limits). The shaded region indicates a 95% confidence interval.

The only index of climate vulnerability that was correlated with survival was CT_max_, with higher CT_max_ associated with lower survival in the days following the experiment (figure 3; electronic supplementary material, table S1). Upon removal of three outliers, the *p*-value for CT_max_ changed from 0.033 to 0.059, indicative of a trend. None of the other indices were correlated with survival (electronic supplementary material, table S1). For *B. impatiens*, the thermal sensitivity of metabolic rate and thermal sensitivity of evaporative water loss were correlated as well as the thermal sensitivity of metabolic rate and the change in evaporative water loss rate (electronic supplementary material, table S2). In both cases, these indices were positively correlated with one another. For *B. auricomus*, the thermal sensitivity of water loss and change in metabolic rate were correlated.

## 4. Discussion

In this study, we evaluated multiple indices of climate vulnerability in two species of bumblebee. We found clear evidence that neither species exhibited acclimation in any of the measured traits. In terms of vulnerability to warm temperatures, the only commonly used index of vulnerability consistent with our hypothesis was the thermal sensitivity of metabolic rate (lower in *B. impatiens*). Therefore, *B. impatiens* may be less vulnerable to warming because energetic costs in *B. impatiens* do not rise with temperature as quickly as in *B. auricomus*. We also found that *B. impatiens* tolerated colder temperatures than *B. auricomus*; although due to the relatively warm temperatures in our experiment, we likely did not fully test acclimation capacities to cold temperatures [80,81]. The results also suggest that these differences were due to inherent characteristics of the species and not differences in body mass, as species was often significant even after accounting for body mass. Our results suggest that the locally declining species, *B. auricomus,* possesses some intrinsic physiological traits that might make it more vulnerable to climate change. The trend towards lower survival in *B. auricomus* after exposure to extreme temperatures and higher sensitivity of metabolic rate could translate into lower foraging efficiency and/or competitive ability. More broadly, however, the inconsistent patterns that we observed among indices suggest that measuring indices of climate vulnerability in isolation may provide misleading insights into organismal responses to environmental change.

Due to the relative ease of measurement, CT_max_ is one of the most commonly measured indices of climate vulnerability [82]. CT_max_ is not intended to be lethal [14]; however, many studies on insects report high mortality after CT_max_ experiments [15,51]. In many cases, explicit measurements of survival are not included in the experimental design of CT_max_, and in other cases, high mortality appears to be a consequence of the experimental design if individuals are not removed upon experiencing CT_max_ [28]. Here, we demonstrate that survival was negatively correlated with CT_max_, even after accounting for species and mass (electronic supplementary material, table S1). Thus, survival was not a function of the critical thermal limit of the species, but rather their degree of exposure to stressful temperatures. Disentangling this relationship is challenging in a dynamic thermal tolerance assay because individuals with lower survival (i.e. higher CT_max_) experienced hotter temperatures for longer durations compared to bees with higher survival. Thus, low survival could be related to experiencing greater physiological damage from experiencing hotter temperatures, expending more energy and/or water during the trial or sustaining greater heat injury at sublethal temperatures. Experiments on lethal limits or survival time (e.g. thermal death time curves) would help to disentangle these issues [83–85]. Regardless, our results suggest that both species began to accumulate physiological damage from heat at the same rate prior to reaching CT_max_ (figure 3B). Thus, we argue that measurements of survival after heat exposure may provide important information about the long-term impacts of extreme heat exposure, thereby improving our understanding of whole-organism performance to environmental change.

Our study also provides potentially novel indices of climate vulnerability for insects that use DGE. Our analysis revealed unique patterns of respiration between the two species that were not captured by mean differences (electronic supplementary material, figure S4). In our experiments, respiration in *B. impatiens* was marked by clear patterns of DGE in both temperature treatments, whereas respiration in *B. auricomus* became more cyclical and shallower (i.e. lower volume per gas exchange cycle) when measured at 30°C compared to measurements at 18°C. The inability to regulate DGE at warm temperatures might help to explain the lower survival after CT_max_ trials in *B. auricomus*. Failure to maintain DGE could have been the result of energy imbalance, protein denaturation or neural damage [86]. If DGE evolved to reduce energetic costs in hot temperatures [87,88], then these species-specific differences in ventilation patterns might provide insight into (or an alternative index of) climate vulnerability within insects.

We did not find any evidence for thermal acclimation in metabolic rate, water loss rate or thermal tolerances in either species. Although we found individual variation in the change of these physiological traits (electronic supplementary material, table S2), the variation was not associated with the acclimation treatment (i.e. current versus future climate). We also found that individuals from Ames exhibited lower water loss rates compared to individuals from Coon Rapids, but our experiments cannot determine whether these differences are due to plastic responses or local adaptation. This evidence suggests that the potential for acclimation capacity to predict species’ climate vulnerability may be especially limited in bumblebees. There are several explanations for the lack of acclimation. First, our experiments may not have been sufficiently extreme to elicit acclimation. However, we doubt this explanation due to the lack of thermal acclimation in similar experiments on bumblebee workers in more extreme temperatures [51,52] and recent evidence that bumblebees experience severe physiological stress (and high mortality) at temperatures between 32°C and 35°C [55]. Second (and more likely), natural selection may not have favoured the evolution of thermal acclimation to warm temperatures in bumblebees. For acclimation to evolve, individuals must be exposed to reliable cues that predict future environmental conditions [89]. Bumblebee queens are generally active in ambient conditions for the first few weeks of nest initiation, but once the nest has produced sufficient workers, queens spend most of their time inside the nest performing reproductive activities [90]. Most individuals (including workers) also cease activity during very hot or cold temperatures and are thus buffered from extreme fluctuations in their underground nests [62,91]. Such avoidance behaviour could buffer bumblebees from selection on acclimation capacity (i.e. the Bogert effect) and thus limit evolutionary responses to future warming [92]. Avoidance of such costs may improve survival in the short term but will presumably have costs for energy balance and reproduction over time [93]. In the absence of acclimation, alternative indices of climate vulnerability may need to be considered.

Our study demonstrated few correlations between indices of climate vulnerability and survival. The only index correlated with survival was CT_max_, with higher CT_max_ being associated with lower survival. One explanation may be that the neurological controls driving locomotion during thermal tolerance experiments are different from the processes underlying longer term survival. For instance, neurological failure in the brain does not appear to underlie thermal tolerances [94], but perhaps plays a role in longer term survival after exposure to extreme temperatures. In addition, we found relatively few associations among indices of climate vulnerability (electronic supplementary material, table S2). The only associations occurred between aspects of metabolic rate and water loss rates. The correlations between metabolic rate and water loss rate could reflect linkages driven by the requisite loss of water during respiration [95]. Given the general lack of associations among these indices in our study and others [96], a broader taxonomic study might reveal the various life history, evolutionary or context-specific factors shaping the linkages between these indices across taxa. Nevertheless, our study calls for more comprehensive experiments on multiple aspects of thermal performance and survival to understand the relationship among indices of climate vulnerability across taxa.

Understanding the physiological mechanisms underpinning species’ climate vulnerability is critical for developing more accurate predictions of the ecological impact of climate change. Our results also emphasize the importance of understanding the relationship between survival at different time scales and indices of climate vulnerability, such as CT_max_. Our findings suggest that the thermal physiology of each species consists of multiple, independent traits that have not necessarily undergone correlated evolution, and thus we should not expect a single species to be ‘vulnerable’ across the board for all indices. We encourage the measurement of multiple indices in concert to gain a more complete understanding of which aspects of climate may be most challenging to a given organism. Our study also underscores the need to evaluate whether these physiological indices are correlated to survival or performance in nature, particularly during and after exposure to hot conditions. Thus, whether CT_max_ or other indices predict responses to climate change in isolation remains poorly understood. Such experiments that explore these dynamics will improve our understanding of whole-organism performance as well as the mechanisms driving climate vulnerability.

## Data Availability

Data for the study can be found on Figshare [97]. Supplementary material is available online [98].
